# Three Cases of Puerperal Convulsions Treated by Venesection

**Published:** 1883-07

**Authors:** J. Fuller

**Affiliations:** Irel., Long Ashton


### THREE CASES OF PUERPERAL CONVULSIONS TREATED BY VENESECTION. By J. Fuller, M.K.Q.C.P., Irel., Long Ashton.

I.—Mrs. S., age 26, was delivered of her first child on May 26th, at 6 p.m., and was attended by Mr. Ormerod, of Westbury. She had a natural labour, and there was nothing to indicate that convulsions might occur; she was seen by him about 5 p.m. the next evening, and appeared to be doing well. About 6.30 the same evening a messenger came to ask him to come and see her again, as she had had three fits since he left her. He kindly asked me to accompany him. On arrival we found she had had two more fits since the messenger started, but was then free from them. Her face was slightly puffed, and, on drawing off some of her urine, it was found to be highly albuminous. Her pulse was full and strong. She was given an enema of turpentine, and, there being no return of the convulsions, we left. We had not been home more than two hours when we were sent for again, as the convulsions had returned. Mr. Ormerod having an engagement asked me to go and see her, and suggested that if other means failed I should bleed her. On my arrival I found her unconscious; she had had two fits since we left her, but had not recovered consciousness from the last; her breathing was slow and laboured, pulse full and slow, pupils dilated and insensible. I gave her an injection of 40 grains of chloral and the same of bromide of potassium, which had no effect. She continued having the fits every half hour, but did not return to consciousness between them. As I had waited about five hours and there was no prospect of improvement I took 35 oz. of blood rapidly from her arm, she appeared quiet for three-quarters of an hour, but did not regain consciousness. The fits returning I removed the bandage and took 10 oz. more. The fits ceased and she became conscious within an hour. There was no return of them. Next day I found her doing well, but complaining of pain in her left shoulder. Examination revealed dislocation of the humerus, downwards into the axilla, done by muscular action alone. This Mr. Ormerod reduced. She made a good recovery.

II.—The second occurred in my own practice at Long Ashton. On March 2nd, 1882, I was sent for to attend Mrs. H., who expected to be confined of her third child. On arrival I found that labour had commenced at 7 a.m.; the pains had been feeble and irregular until 8 p.m. when they became more severe. The nurse informed me that the patient was convulsed with each of the last five pains, and remained unconscious for some short time after the pains had left her. There was then an interval of some twenty minutes between each pain. There was no swelling of the face, but the hands were somewhat swollen; no albumen in the urine; she had a fair pulse. On examination I found the os about the size of a fiveshilling piece, hard and undilatable, the head presenting, a pain then coming on the woman was again convulsed. The nurse remarked that it was worse than the previous ones, as it lasted longer, and the return to consciousness was not so complete as before. The pains kept increasing in frequency, and with them the convulsions. There was now no return to consciousness between the attacks. I tried the inhalation of chloroform, which certainly diminished the violence of the attacks, but did not quite stop them, and when I left off using it they returned as violent as ever. As I had now waited more than three hours, and though the pains were very strong, there was no perceptible dilatation of the os, I began to get anxious about her, and decided on trying venesection. I took 30 oz. of blood rapidly from her arm, the convulsions ceased almost immediately, the os became soft and dilatable, and in less than an hour I was able to deliver with the long forceps. The child was dead, but the mother made a good recovery.

III.—The third case, Mary G., unmarried, was delivered of her second child on August 8th, 1882, at 11 a.m. She had, I understood, a natural labour. Twelve hours after she was seized with convulsions, which she continued to have with very slight intermissions until four next morning, when I was sent for. She was unconscious when I arrived, and her mother told me she had been like that for an hour, and she had had three fits in that time; her face and legs were œdematous, urine albuminous, pupils dilated, pulse rather weak. As they had not informed me of the nature of the case before I started I had nothing with me to give her. Being some distance from home I did not like to leave her. Having no one to send for anything, the fits continuing, though not violent, and consciousness not returning, I decided on bleeding, and took, I should say, from 25 to 30 oz. (I had nothing to measure it with) of blood from her; the fits entirely ceased, though she did not become quite conscious for some hours after. She made a somewhat slow recovery. As she had the swelling of the face and legs prior to her first confinement, but no convulsions, it did not give her any uneasiness.

In treating these cases I held these points of pre-eminent importance—to take away plenty of blood and to take it rapidly.

